# The association between eating difficulties and biliary sludge in the gallbladder in older adults with advanced dementia, at end of life

**DOI:** 10.1371/journal.pone.0219538

**Published:** 2019-07-16

**Authors:** Takahide Miyamoto, Takae Ebihara, Koichi Kozaki

**Affiliations:** Department of Geriatric Medicine, Kyorin University School of Medicine, Tokyo, Japan; University of Alberta, CANADA

## Abstract

**Objectives:**

In clinical settings, untreatable biliary sludge in the gallbladder can be observed in older adults with advanced dementia. The underlying cause of biliary sludge existence in patients with dementia is currently unknown. Therefore, we aimed to investigate the prevalence, risk factors, and related outcomes of biliary sludge formation in the gallbladder of older adults with dementia.

**Design:**

Cross-sectional study.

**Setting:**

Geriatric ward of University Hospital in Japan.

**Participants:**

Inpatients aged 80 and older living with dementia.

**Measurements:**

We evaluated the presence of biliary sludge by diagnostic ultrasonography and collected data regarding patient demographic information, cognition (mini-mental state examination [MMSE]), physical activity (Barthel Index), oral food intake (food intake level scale [FILS]), clinical stage of dementia (functional assessment staging [FAST] of dementia), and patient performance status (Zubrod/ Karnofsky score).

**Results:**

Male sex, larger gallbladder volume and calories from oral intake were significantly associated with the presence of biliary sludge (*P* = .02, .02, .002, respectively). There was a significant negative correlation between the FAST stage and the FILS level in all patients (*P* < .001). More advanced dementia and dysphagia was more likely to be found in patients with Alzheimer disease (AD) with biliary sludge, compared to patients with AD without biliary sludge (FAST 7a, FILS II and FAST 6c, FILS V, respectively, *P* = .06, 04). A logistic regression analysis revealed that the eating status of FILS I and II, generally called “fasting or anorexia”, was a significant risk factor for forming biliary sludge in older adults with dementia (*P* = .031, odds ratio: 5.25, 95% confidence interval: 1.16–23.72).

**Conclusions:**

Fasting status may be associated with the existence of biliary sludge in older adults with dementia. Therefore, supportive care for eating might be an important solution to comfortable end-of-life care for older adults with advanced dementia.

## Introduction

Many older adults with advanced dementia have eating difficulties [1, 2]. These are often due to having a swallowing disorder (dysphagia) or decreased appetite or refusing to eat or to drink. For the patient, this can result in aspiration pneumonia and malnutrition. Therefore, as the stage of dementia advances, many older adults with dementia require parenteral nutrition. Although difficulty eating, or swallowing can be representative symptoms of end of life in dementia, an important aspect of palliative care—particularly for the concerned family members of patients—is a better understanding of how to provide supportive care for eating. However, the relationship between the clinical stages of dementia and dysphagia, and consequent symptoms or diseases, has not yet been understood.

A previous nursing home study reported that half of the residents with advanced dementia had pyrexia, pneumonia, or died within a year and a half after their admission [2]. There is limited evidence of the incidence of cholangitis or cholecystitis as the cause of pyrexia or death in patients with advanced dementia. However, in a clinical setting, we have often noted biliary sludge in the gallbladder of older adults with advanced dementia. In previous studies, the conventional causes of biliary sludge have been reported as pregnancy, rapid weight loss, total parenteral nutrition, octreotide therapy, and other factors unrelated to aging or dementia [3, 4].

Severe biliary sludge is often associated with the occurrence of cholangitis or cholecystitis, which results in the need for percutaneous transhepatic biliary drainage (PTBD) or percutaneous transhepatic gallbladder drainage (PTGBD). Age and immobility of the gallbladder due to fasting have been found to promote biliary sludge formation [4]. However, despite the rapidly growing population of older adults with dementia, there are no studies focusing on factors related to biliary sludge of the gallbladder in this population. Therefore, the purpose of this study was to investigate the prevalence, risk factors, and related outcomes of the formation of biliary sludge of the gallbladder in older adults with dementia.

## Methods

This study included older adults with dementia who were admitted to the geriatric ward of University Hospital in Japan between May and November 2016. Our inclusion criteria were persons living with dementia more than 65 years, admitted to the geriatric ward who complained of a decreased appetite. Dementia was diagnosed by doctors who have specialist qualification of dementia. Differentiation of dementia diagnosis is described in the next section. Our exclusion criteria were persons with a disturbance of consciousness, admitted to the intensive care unit and fed via gastrostomy. Ultrasonography was used to calculate the volume of the gallbladder and assess the existence of biliary sludge in the gallbladder. The volume of the gallbladder was estimated as an ellipsoid using a formula of V=4πabc/3 (V: volume, a: semilength of width, b: semilength of height, c: semilength of depth). Ultrasonography was performed while patients were fasting before a meal after hospitalization. Laboratory blood analysis included serum total cholesterol, serum low-density lipoprotein cholesterol, serum total bilirubin, and serum albumin-adjusted calcium. Patients’ cognition and clinical stage of dementia were evaluated using the mini-mental state examination (MMSE) [5,6,7] (S1 Table) and the functional assessment staging (FAST) classification [8,9] (S2 Table). Patients’ individual level of activities of daily living (ADL) was assessed using the Barthel Index [10,11] (S3 Table), and performance status was assessed using the Zubrod (Karnofsky) performance scale [12,13] (S4 Table). Additionally, we evaluated the patients’ ability to eat using the Food Intake Level Scale (FILS) [14] (S5 Table) and investigated calories of oral food intake from each patient’s medical record. In patients who experienced a low appetite and/or poor oral intake (FILS score less than VI), diagnostic imaging such as videoendoscopy, videofluoroscopy, gastroendoscopy, computed tomography, and echocardiography to examine the pharynx, the larynx and the gastrointestinal tract were performed; in addition, blood tests and urinalyses were done to rule out cancer, metabolic problems, liver disease, respiratory disease, hypothyroidism, chronic kidney failure and heart failure. The results of these tests indicated no clear cause of the low appetite except dysphagia in older adults with dementia.

This study was approved by the ethics committee of Kyorin University School of Medicine (Approval number: 28–008), and informed consent was obtained from all participants or family members in cases where the patients did not have the capacity and were diagnosed as a dementia using the MMSE. This consent procedure was approved by our ethics committee.

### Differentiation of dementia diagnosis

Dementia was diagnosed based on cognitive decline, as measured by the neuropsychological test battery and observation of impairments in social or occupational functioning. Alzheimer disease (AD) and vascular dementia (VaD) were diagnosed according to the NINCDS-ADRDA and NINDS-AIREN criteria [15, 16, 17] each in combination with cerebral imaging, judged by at least one doctor who has specialist qualification of dementia. We used brain magnetic resonance imaging to investigate structural changes in the brain tissue. Specifically, a voxel-based specific regional analysis system (Eisai Co, Ltd, Tokyo, Japan) was used to examine the degree of atrophy in the hippocampus during the diagnostic process for patients with AD [18]. Brain single-photon emission computed tomography was also used, as it detects changes in the bloodstream in specific regions of the brain and can thus be useful in the diagnosis of AD or VaD.

### Evaluation of body mass index (BMI)

For better accuracy when measuring the height and weight of older adults with kyphosis or those who had difficulty standing, we calculated patients’ height using a knee height caliper [19], and their weights were measured while they were lying down. Height and weight measurements were used to calculate patients’ body mass index (BMI).

### Data statistics

To compare patients with and without biliary sludge, we used the Mann–Whitney U test to analyze the influence of age, serum total cholesterol, serum low-density cholesterol, serum total bilirubin, albumin-adjusted calcium, the MMSE, the Barthel Index, calories from oral intake, BMI, and volume of the gallbladder.

We used the Fisher exact test to analyze the sex of the participants and the number of patients with dementia. The number of participants with a gallstone, the number of participants medicated with an anticholinergic agent, and the number of participants that had cholangitis or cholecystitis were analyzed with the Fischer exact test. Spearman’s rank correlation test was used to analyze the correlation between FAST stage and FILS level, and to calculate the central median value in the subclassified groups in the FILS, the Barthel Index, the FAST, and the Zubrod/Karnofsky score. Multivariate logistic regression analysis was performed to estimate the odds ratio (ORs) and 95% confidence intervals (CIs) of the relationship between the existence of biliary sludge and age, sex, the cutoff FILS level (II/III), the cutoff Barthel Index (40/41), the cutoff FAST stage (6c/6d), and the cutoff Zubrod/Karnofsky score (2/3). The sample size in the study was estimated as 48 (statistical power was determined by the following variables: α, 0.05 and β, 0.2, effect size | r | 0.5, disease prevalence and controls in this study were 24.4% and 74.8%, respectively). A *P*-value < 0.05 was deemed statistically significant. Data were expressed as mean (SD) except where specified otherwise. All data were analyzed using SPSS version 22.0 (IBM Corp., Tokyo, Japan). Sample size was calculated by G*Power 3.1.9.2.

## Results

A total of 123 older adults (mean age: 87.6 ± 5.3 years) were enrolled in this study. Of these, 30 patients had biliary sludge, while 93 did not have biliary sludge. Patients’ characteristics are described in Table 1. Between the two groups of patients with and without biliary sludge, no significant differences were found related to age, BMI, serum total cholesterol, serum low-density cholesterol, serum total bilirubin, albumin-adjusted calcium, the MMSE, type of dementia (e.g. AD or VaD), the Barthel Index, calories of oral food intake, or number of patients with gallstone, cholangitis or cholecystitis. Male patients had more biliary sludge than female patients (19 [63.3%] versus 11 [36.7%], respectively) (*P* = .02). The volume of the gallbladder in patients with biliary sludge was greater than that in patients without biliary sludge (29.74 ±25.42 cm^3^ versus 13.29 ± 13.81 cm^3^, respectively, *P* = .002). The number of patients receiving anticholinergic agents in the group without biliary sludge was greater than that in the group with biliary sludge. (19 [20.4%] versus 1 [3.4%], *P =* .04).

**Table 1:** Patient characteristics.

	Biliary sludge (-)	Biliary sludge (+)	*P* value
Number of participants	93	30	
Age (± SD)	87.8 (± 5.2)	87.0 (± 5.4)	0.44
Male sex, N (%)	36(38.7)	19 (63.3)	0.02
Body Mass Index	20.58(± 4.38)	20.37 (± 4.18)	0.82
Calories from oral intake (± SD) (kcal)	786.02 (± 561.75)	503.33(± 583.97)	0.02
MMSE (± SD)	12.99 (± 8.67)	11.97 (± 10.03)	0.59
Number of patients with dementia AD/VaD, N	33/60	11/19	1.00
Barthel Index (± SD)	39.033 (± 31.69)	43.67 (± 34.19)	0.50
Volume of the gallbladder (cm^3^)	13.29 (± 13.81)	29.74 (± 25.42)	0.002
Serum total cholesterol mg/dl	155.05(±45.56)	137.71(±34.66)	0.15
Serum low-density lipoprotein cholesterol (LDL) mg/dl	84.87 (± 34.31)	68.81 (± 23.26)	0.01
Serum total bilirubin mg/dl	0.77 (± 0.48)	0.73 (± 0.56)	0.72
Serum albumin-adjusted calcium mg/dl	9.53 (± 0.53)	9.38 (± 0.44)	0.17
Number of patients with cholelithiasis N	16	5	1.00
Number of patients with anticholinergic agent N	19	1	0.04
Number of patients with cholecystitis and cholangitis N	1	0	1.00

Age, calorie intake, MMSE, Barthel Index, and the volume of the gallbladder were statistically analyzed using the Mann–Whitney U test.

Sex, the number of patients with dementia, cholelithiasis and anticholinergic agent, and the number of patients with cholecystitis and cholangitis, were analyzed using the Fischer exact test.

*P* < 0.05 was statistically significant.

*Abbreviations*: MMSE, mini-mental state examination; LDL, low density lipoprotein

**Fig 1:**
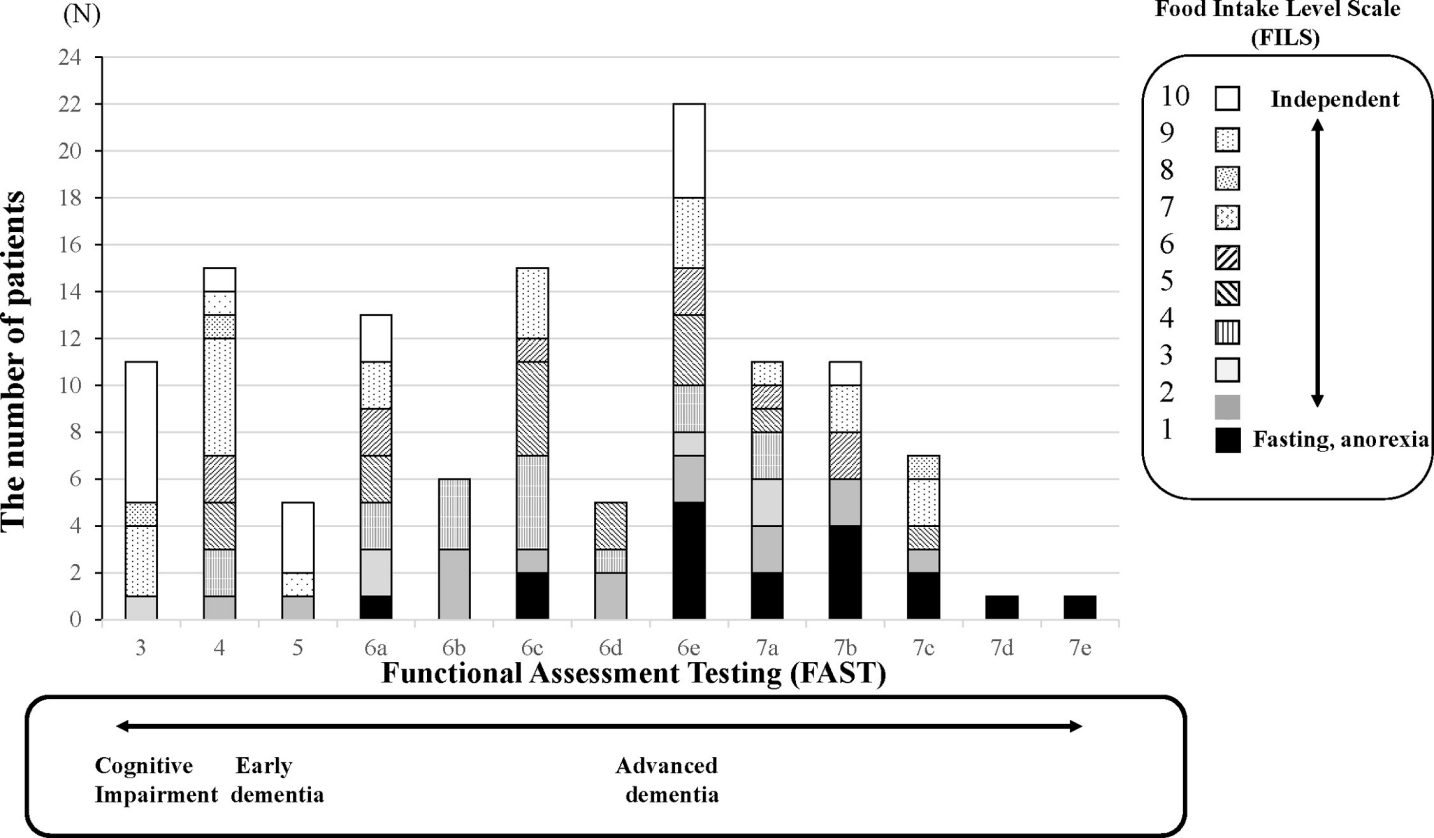
Relationship between the FAST level and the FILS level in patients with dementia. These figures showed the relationship between the FAST level and the FILS level in all patients. Each bar on the FAST level was expressed as accumulation of the number of persons in each level of the FILS. Spearman’s rank correlation test showed significant correlations between the FAST level and the FILS level in all patients (R = -0.414, *P* < .001). Abbreviations: FILS, food intake level scale; FAST, functional assessment staging.

The relationship between the FAST stage and the FILS level showed a significant negative correlation in all enrolled patients (r = -0.414, *P* < .001) (Fig 1). In particular, the significance of the negative correlation between the FAST and the FILS was more evident in patients with AD (r = -0.66, *P* < .001) than patients with VaD (r = -0.24, *P* = .03).

In all patients, the FILS stage in patients with biliary sludge tended to be worse than that in patients without biliary sludge (central median value in patients with biliary sludge versus without biliary sludge: FILS III versus FILS VI, *P* = .15) (Tables 2 and 3). Patients with AD with biliary sludge showed a worse FAST stage and FILS level than those without sludge (FAST 7a, FILS II versus FAST 6c, FILS V; *P* = .06, .04, respectively). Similarly, patients with VaD with biliary sludge were generally in a worse FILS level compared to patients with VaD without biliary sludge (FILS V versus FILS VI, *P* = .11).

**Table 2:** Central median value of FAST, FILS, Barthel Index and Zubrod score.

	Central Median Value			
	FAST	FILS	Barthel Index	Zubrod/Karnofsky score
All Participants	6c	V	40	2
AD	6e	V	20	2
VaD	6c	VI	45	2

*Abbreviations*: AD Alzheimer’s disease; VaD, vascular dementia; FILS, food intake level scale; FAST, functional assessment staging

**Table 3:** Comparison of a central median value of FAST, FILS, Barthel Index and Zubrod score in patients with biliary sludge and without biliary sludge.

Variables	Central Median Value											
	FAST			FILS			Barthel Index			Zubrod/Karnofsky score		
	Biliary Sludge (+)	Biliary Sludge (-)	*P* value	Biliary Sludge (+)	Biliary Sludge (-)	*P* value	Biliary Sludge (+)	Biliary Sludge (-)	*P* value	Biliary Sludge (+)	Biliary Sludge (-)	*P* value
All Participants	6d	6c	0.57	III	VI	0.15	40	40	0.89	2	2	0.50
AD	7a	6c	0.06	II	V	0.04	10	20	0.584	3	2	0.47
VaD	6c	6c	0.853	V5	VI	0.11	45	60	0.16	2	2	0.95

*Abbreviations*: FILS, food intake level scale; FAST, functional assessment staging; AD, Alzheimer disease; VaD, vascular dementia

The central median value of the Barthel Index was 40 in all patients with biliary sludge, but this was not significantly different compared to all patients without biliary sludge (Table 3).

A logistic regression analysis, after adjusting for age, sex and the number of patients administered an anticholinergic agent, using the central median value of the FAST stage, the FILS level, the Barthel Index, and the Zubrod /Karnofsky score, showed that a FILS level less than II was a significant risk factor for the formation of biliary sludge in older adults with dementia (ORs: 5.25, 95% confidence interval: 1.16–23.72, *P* = .03, adjusted for age, sex, and use of anticholinergic agents) (Table 4).

**Table 4:** Logistic regression analysis using the central median value, adjusted for age, sex, and use of anticholinergic agents.

Adjusted for sex, age, and use of anticholinergic agent				
	*P* value	ORs	95% CI	
Fasting	0.03	5.25	1.16	23.72
Clinical stage of dementia	0.54	0.63	0.15	2.72

ORs: odds ratio, CI: confidential interval

## Discussion

To the best of our knowledge, this study is the first report to show the prevalence and risk factors of biliary sludge of the gallbladder in older adults with dementia. Our findings showed that patients with more severe clinical symptoms also showed more limited ability to eat. Additionally, we found that FILS level I and II may be associated with the existence of biliary sludge in older adults with dementia, and that biliary sludge tended to appear in both patients with AD and with VaD that had a fasting status.

According to previous reports, the prevalence of biliary sludge was reported to be 1.7% when found during annual medical examinations in asymptomatic patients, and approximately 7.0% in patients who underwent examinations on account of abdominal symptoms [20]. Conventional causes of the formation of biliary sludge have been reported as pregnancy, rapid weight loss, and total parenteral nutrition [3, 4]. However, although dementia may be associated with rapid weight loss and total parenteral nutrition, no research to date has focused on associations between biliary sludge, dysphagia, and advanced dementia.

In the clinical setting of our institution, we have often encountered patients with advanced dementia who could hardly eat, and who then developed biliary sludge within a few weeks. In these patients, PTBD or PTGBD is often required. Basically, PTGB and PTGBD are temporary installed until the execution of surgery, such as cholecystectomy. In non-geriatric patients, these tubes can be smoothly removed when surgery is completed and postoperative status, evaluated. On the other hand, PTBD or PTGBD execution could be implemented as alternative procedures in patients with advanced dementia who have difficult in eating and are not indicated for surgery. However, removing the sludge through PTBD or PTGBD tube by saline washing each day is usually difficult due to the high viscosity of the biliary sludge and is not an effective treatment option. These experiences influenced the current research, which was undertaken in an effort to better understand how the status of eating correlated to the clinical stage of dementia and the existence of biliary sludge. FILS levels I and II, which correspond to an inability to swallow food [14], were regarded as “fasting or anorexia status” in this study; we found that patients with FILS level I and II were more likely to develop biliary sludge. Messing reported in non-geriatric study that the percentage of sludge positive patients during parental nutrition without oral or enteral routes, increased from 6% during the first 3 weeks to 50% between the fourth and the sixth weeks and reached 100% in patients receiving i.v. nutritional therapy more than 6 weeks. Interestingly, sludge positively decreased from approximate 90% during the first 3 weeks of oral refeeding to 0% by the end of the fourth week [21]. The previous report strongly supports our results in the study.

Our findings show that the ability to eat of a patient with dementia has a more significant correlation with the clinical stage of dementia in patients with AD, compared to patients with VaD. However, FAST is a more reliable assessment tool to evaluate functional deterioration in patients with AD throughout the entire course of the illness. Thus, in patients with AD with a FAST stage of more than 7a, clinicians should focus on the patients’ status of eating and the possible presence of biliary sludge.

In another comparison of demographic factors in patients, the biliary sludge in males and in patients with VaD appeared at the earlier stage of each evaluation scale (Tables 1, 2 and 3). A possible explanation for this would be the patient’s baseline characteristics associated with VaD such as changes in metabolic factors promoting arteriosclerosis.

Compared with a previous report in a non-geriatric population, in which risk factors for gallstones and biliary sludge formation were dyslipidemia, female sex and obesity, possible demographic factors of the existence for biliary sludge formation in this study were not hyperlipidemia and high BMI, but male sex [22]. We speculate that the risk factors for the existence of biliary sludge in geriatric people with dementia might be different from those in non-geriatric people.

Furthermore, comparisons of physical function in patients with AD and VaD in this study did not show a significant effect of the presence of biliary sludge on the Barthel Index or Zubrod/Karnofsky performance status. However, these scores were worse in patients with AD with biliary sludge (average Barthel Index total score: 10, average performance status: 3), compared to in patients with VaD and biliary sludge (average Barthel Index total score: 45; average Zubrod/Karnofsky performance status, 2) (Table 3). In general, functional gallbladder disorder is diagnosed by Rome III criteria [23]. However, it is difficult to diagnose functional gallbladder disorder in people with dementia because they have difficulty to complain symptoms. Although the gallbladder in patients with AD is likely immobile or frail, deterioration of physical function and performance status did not significantly correlate with the existence of biliary sludge in this study. In addition, in our clinical experience, patients with biliary sludge have ceased to eat within a few weeks of admission, whereas patients without biliary sludge continue to eat, even if only in small amounts.

This study has some limitations. We could not compare our cohort to a control group of non-geriatric patients which may have helped the validity of our result; however, the occurrence of dysphagia among non-geriatric people is quite limited and any occurrence is generally associated with a smooth recovery. Additionally, function of the gallbladder among non-geriatric patients is generally more efficient than that of geriatric people. We were unable to determine what caused the onset of biliary sludge formation, nor could we investigate whether acetylcholine esterase inhibitor medication caused biliary sludge due to its possible action on the sphincter of Oddi. In this study, a significant number of patients without biliary sludge were receiving anticholinergic agents. Moreover, we could not evaluate the motility of the gall bladder and evaluate whether biliary sludge was related to the development of cholangitis or cholecystitis.

## Conclusions

In conclusion, fasting status may be associated with the existence of biliary sludge in older adults with dementia, as biliary sludge tends to appear in older adults who have AD or VaD and who are not eating well. If an older adult with dementia enters a fasting state, clinicians should consider the patient’s gallbladder in addition to monitoring oral intake status. The presence of biliary sludge, in patients with advanced dementia, may be a sign that the end of life is near. As fasting in older patients with advanced dementia appears to be associated with the development of biliary sludge of the gall bladder, it is important to develop appropriate and sufficient supportive care to help older adults with dementia improve their ability and willingness to eat. The results of this study suggest that palliative care is the most appropriate next step for patients to transition to the end of life in comfort.

## Supporting information

S1 Table The mini-mental state examination (MMSE). Evaluation of cognitive function- [5, 6, 7]. Reliability (test-retest); sensitivity 81%, Specificity 89%[6]. Predictive validity of MMSE-J (the serial 7s version) sensitivity 0.86, specificity 0.89, The test-retest reliability 0.81) Internal consistency reliability showed Cronbach alfa coefficient 0.58 [7].
(DOCX)

S2 Table The functional assessment staging (FAST) classification. -Evaluation of clinical stage of dementia- [8, 9]. Reliability; rater consistency (fixed effect ICC) was 0.86 and rater agreement (random effect ICC) was 0.87. Correlation coefficients between FAST levels and the individual the ordinal scales of psychological development (OSPD) was 0.79[9].
(DOCX)

S3 Table The Barthel index. -Evaluation for Activities of Daily Living—[10,11]. Inter-rater reliability: ICC 0.89, Reliability(test-retest):ICC 0.95–0.97 [11]
(DOCX)

S4 Table The Zubrod (Karnofsky) performance scale. -Evaluation of performance status- [12.13]. Inter-rater Reliability:Pearson Correlation-R 0.89, Kappa Statistic 0.53[13]
(DOCX)

S5 Table The food intake level scale (FILS). -Evaluation of ability of eating and drinking—[14]. Inter-rater reliability 0.83–0.90, weighted kappa coefficients 0.70 to 0.90 [14]
(DOCX)

## References

[1] Livingston G, Sommerlad A, Orgeta V, Costafreda SG, Huntley J, Ames D, et al Dementia prevention, intervention, and care. Lancet 2017; 390: 2673–2734 10.1016/S0140-6736(17)31363-6 28735855

[2] Mitchell SL, Teno JM, Kiely DK, Shaffer ML, Jones RN, Prigerson HG, et al The clinical course of advanced dementia. New Engl J Med 2009; 361: 1529–1538. 10.1056/NEJMoa0902234 19828530 PMC2778850

[3] Pazzi P, Gamberini S, Buldrini P, Gullini S. Biliary sludge: the sluggish gallbladder. Dig Liver Dis 2003;35(Suppl 3): S39–S45. 12974509 10.1016/s1590-8658(03)00093-8

[4] Angelico M, De Santis A, Capocaccia L. Biliary sludge: a critical update. J Clin Gastroenterol. 1990; 12: 656–662. 2266242 10.1097/00004836-199012000-00012

[5] Folstein MF, Folstein SE, McHugh PR. “Mini-mental state”: a practical method for grading the cognitive state of patients for the clinician. J Psychiatr Res. 1975; 12: 189–198. 1202204 10.1016/0022-3956(75)90026-6

[6] Stuss DT, et al Do long tests yield a more accurate diagnosis dementia than short tests? A comparison of 5 neuropsychological tests, Archives of Neurology 1996;53;1033–1039 8859066 10.1001/archneur.1996.00550100119021

[7] Sugishita M, Hemmi I, JADNI. Validity and Reliability of the Min Mental State Examination-Japanese (MMSE-J): A Preliminary Report. Japanese Journal of Cognitive Neuroscience 2010; 12: 186–190.

[8] Reisberg B. Functional assessment staging (FAST) Psychopharmacol Bull. 1988; 24: 653–659. 3249767

[9] Sclan SG, Reisberg B. Functional assessment staging (FAST) in Alzheimer's disease: reliability, validity, and ordinality. Int Psychogeriatr. 1992; 4 Suppl 1: 55–69. 10.1017/s1041610292001157 1504288

[10] Mahoney FI and Barthel DW. Functional evaluation: the Barthel Index. Md State Med J. 1965; 14: 61–65 14258950

[11] Sainsbury A, Seebass G, Bansal A, Young JB. Reliability of the Barthel Index when used with older people. Age and Aging 2005; 34: 228–232. 10.1093/ageing/afi063 15863408

[12] Oken MM, Creech RH, Tormey DC, Horton J, Davis TE, McFadden ET, et al Toxicity and response criteria of the Eastern Cooperative Oncology Group. Am J Clin Oncol. 1982; 5: 649–655. 7165009

[13] Schag CC, Heinrich RL and Ganz PA. Karnofsky Performance Status Revisited: Reliability, Validity, and Guidelines. J Clin Oncology 1984; 2: 187–193 10.1200/JCO.1984.2.3.187 6699671

[14] Kunieda K, Ohno T, Fujishima I, Hojo K, Morita T. Reliability and validity of a tool to measure the severity of dysphagia: the Food Intake LEVEL Scale. J Pain Symptom Manage. 2013; 46: 201–206. 10.1016/j.jpainsymman.2012.07.020 23159683

[15] McKhann G, Drachman D, Folstein M, Katzman R, Price D, Stadlan EM. Clinical diagnosis of Alzheimer's disease: report of the NINCDS-ADRDA Work Group under the auspices of Department of Health and Human Services Task Force on Alzheimer's Disease. Neurology. 1984; 34: 939–944. 10.1212/wnl.34.7.939 6610841

[16] Róman GC, Tatemichi TK, Erkinjuntti T, Cummings JL, Masdeu JC, Garcia JH, et al Vascular dementia: diagnostic criteria for research studies: report of the NINDS-AIREN International Workshop. Neurology. 1993; 43: 250–260. 10.1212/wnl.43.2.250 8094895

[17] Diagnostic and Statistical Manual of Mental Disorders: 5th edition. American Psychiatric Association, Washington, 2013; 621–624. 10.1108/RR-10-2013-0256

[18] Hirata Y, Matsuda H, Nemoto K, Ohnishi T, Hirao K, Yamashita F, et al Voxel-based morphometry to discriminate early Alzheimer's disease from controls. Neurosci Lett. 2005; 382: 269–274. 10.1016/j.neulet.2005.03.038 15925102

[19] Chumlea WC, Roche AF, Steinbaugh ML. Estimating stature from knee height for persons 60 to 90 years of age. J Am Geriatr Soc. 1985; 33: 116–120. 10.1111/j.1532-5415.1985.tb02276.x 3968366

[20] Janowitz P, Kratzer W, Zimmer T, Tudyka J, Wechsler JG. Gallbladder sludge: spontaneous course and incidence of complication in patients without stones. Hepatology. 1994; 20: 291–294. 8045489

[21] Messing B, Bories C, Kunstlinger F, Bernier JJ. Does total parentetal nutrition induced gallbladder sludge formation and lithiasis? Gastroenterology 1983; 84: 1012–1019. 6403401

[22] O’Connell K and Brasel K. Bile metabolism and lithogenesis. Surg Clin N Am 2014;94:361–375. 10.1016/j.suc.2014.01.004 24679426

[23] Drossman DA. Rome III: the new criteria. Chin J Dig Dis 2006;7:181–185. 10.1111/j.1443-9573.2006.00265.x 17054578

