# Supplementary material for: The association between eating difficulties and biliary sludge in the gallbladder in older adults with advanced dementia, at end of life
Source: PLoS One. 2019 Jul 16;14(7):e0219538. doi: 10.1371/journal.pone.0219538 (PMC6634396; doi:10.1371/journal.pone.0219538)
Supplement: S1 Table — Evaluation of cognitive function- [5, 6, 7]. Reliability (test-retest); sensitivity 81%, Specificity 89%[6]. Predictive validity of MMSE-J (the serial 7s version) sensitivity 0.86, specificity 0.89, The test-retest reliability 0.81) Internal consistency reliability showed Cronbach alfa coefficient 0.58 [7]. (DOCX) [file pone.0219538.s001.docx]

| **Maximum Score** | **Questions** |
| --- | --- |
| 5 | What is the year? Season? Date? Day of the week? Month?” |
| 5 | “Where are we now: State? County? Town/city? Hospital? Floor?” |
| 3 | The examiner names three unrelated objects clearly and slowly, then asks the patient to name all three of them. The patient’s response is used for scoring. The examiner repeats them until patient learns all of them, if possible. Number of trials: ___________ |
| 5 | “I would like you to count backward from 100 by sevens.” (93, 86, 79, 72, 65, …) Stop after five answers. Alternative: “Spell WORLD backwards.” (D-L-R-O-W) |
| 3 | Earlier I told you the names of three things. Can you tell me what those were?” |
| 2 | Show the patient two simple objects, such as a wristwatch and a pencil, and ask the patient to name them. |
| 1 | “Repeat the phrase: ‘No ifs, ands, or buts. |
| 3 | Take the paper in your right hand, fold it in half, and put it on the floor.” (The examiner gives the patient a piece of blank paper.) |
| 1 | Please read this and do what it says.” (Written instruction is “Close your eyes.”) |
| 1 | Make up and write a sentence about anything.” (This sentence must contain a noun and a verb.) |
| 1 | Please copy this picture.” (The examiner gives the patient a blank piece of paper and asks him/her to draw the symbol below. All 10 angles must be present and two must intersect.) |
| 30 | **TOTAL** |

S1 Table. **The Mini-Mental State Examination (MMSE)**

**-**Evaluation of cognitive function- [5, 6, 7]
