# Supplementary material for: The association between eating difficulties and biliary sludge in the gallbladder in older adults with advanced dementia, at end of life
Source: PLoS One. 2019 Jul 16;14(7):e0219538. doi: 10.1371/journal.pone.0219538 (PMC6634396; doi:10.1371/journal.pone.0219538)
Supplement: S2 Table — -Evaluation of clinical stage of dementia- [8, 9]. Reliability; rater consistency (fixed effect ICC) was 0.86 and rater agreement (random effect ICC) was 0.87. Correlation coefficients between FAST levels and the individual the ordinal scales of psychological development (OSPD) was 0.79[9]. (DOCX) [file pone.0219538.s002.docx]

S2 Table.**The functional assessment staging (FAST) classification**

-Evaluation of clinical stage of dementia- [8, 9]

Reliability; rater consistency (fixed effect ICC) was 0.86 and rater agreement (random effect ICC) was 0.87. Correlation coefficients between FAST levels and the individual the ordinal scales of psychological development (OSPD) was 0.79[9].
