# Supplementary material for: The association between eating difficulties and biliary sludge in the gallbladder in older adults with advanced dementia, at end of life
Source: PLoS One. 2019 Jul 16;14(7):e0219538. doi: 10.1371/journal.pone.0219538 (PMC6634396; doi:10.1371/journal.pone.0219538)
Supplement: S3 Table — -Evaluation for Activities of Daily Living—[10,11]. Inter-rater reliability: ICC 0.89, Reliability(test-retest):ICC 0.95–0.97 [11] (DOCX) [file pone.0219538.s003.docx]

S3 Table. **The Barthel Index**

**-**Evaluation for Activities of Daily Living **-** [10,11]

Inter-rater reliability: ICC 0.89, Reliability(test-retest):ICC 0.95–0.97 [11]
