# Supplementary material for: The association between eating difficulties and biliary sludge in the gallbladder in older adults with advanced dementia, at end of life
Source: PLoS One. 2019 Jul 16;14(7):e0219538. doi: 10.1371/journal.pone.0219538 (PMC6634396; doi:10.1371/journal.pone.0219538)
Supplement: S4 Table — -Evaluation of performance status- [12.13]. Inter-rater Reliability:Pearson Correlation-R 0.89, Kappa Statistic 0.53[13] (DOCX) [file pone.0219538.s004.docx]

S4 Table. **The Zubrod (Karnofsky**) **performance scale**

-Evaluation of performance status- [12.13]

Inter-rater Reliability :Pearson Correlation-R 0.89, Kappa Statistic 0.53[13]
