# Supplementary material for: The association between eating difficulties and biliary sludge in the gallbladder in older adults with advanced dementia, at end of life
Source: PLoS One. 2019 Jul 16;14(7):e0219538. doi: 10.1371/journal.pone.0219538 (PMC6634396; doi:10.1371/journal.pone.0219538)
Supplement: S5 Table — -Evaluation of ability of eating and drinking—[14]. Inter-rater reliability 0.83–0.90, weighted kappa coefficients 0.70 to 0.90 [14] (DOCX) [file pone.0219538.s005.docx]

S5 Table. **The Food Intake Level Scale (FILS)**

-Evaluation of ability of eating and drinking - [14]

Inter-rater reliability 0.83-0.90, weighted kappa coefficients 0.70 to 0.90 [14]
